# Supplementary figures and images for: Crumbs2 mediates ventricular layer remodelling to form the spinal cord central canal
Source: PLoS Biol. 2020 Mar 9;18(3):e3000470. doi: 10.1371/journal.pbio.3000470 (PMC7108746; doi:10.1371/journal.pbio.3000470)

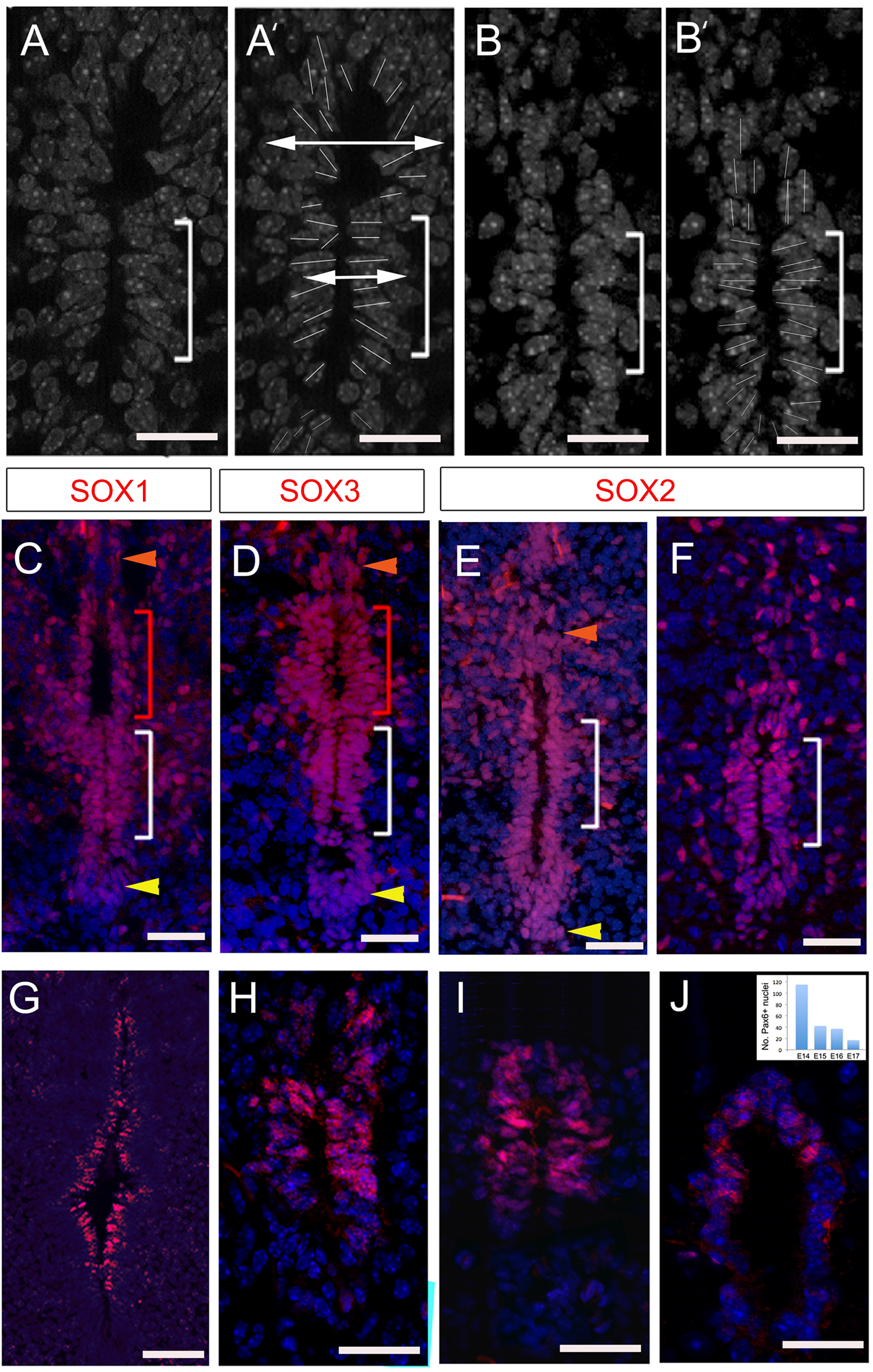

Supplement: S1 Fig — All panels show high-magnification views of the VL in transverse sections at different time points. In (A-F), white bracket demarcates vVL. (A, A′) At E16, DAPI labelling reveals mediolaterally oriented vVL cell nuclei around a narrow lumen, and diagonally oriented dVL cell nuclei around a wider lumen. (B, B′) At E17, dVL cell nuclei are dorsoventrally oriented. (C-F) SOXB1-immunolabelled cells at E15.5 (C,D), E16 (E), and E17 (F). Red bracket demarcates dVL; yellow arrowheads show dissociating floor plate cells; orange arrowheads show excluded SOXB1(+) cells in the dorsal midline. (G-J) Pax6 labelling at E14 (G), E15 (H), E16 (I), and E17 (J). PAX6(+) cells are excluded in dorsal collapse (inset, J). Scale bars: A-F, 50 μm; G-J, 100 μm. dVL, dorsal ventricular layer; PAX6, paired box 6; SoxB1, SRY-related HMG-box B1 transcription factors; VL, ventricular layer; vVL, ventral ventricular layer. (TIF) [file pbio.3000470.s001.tif]

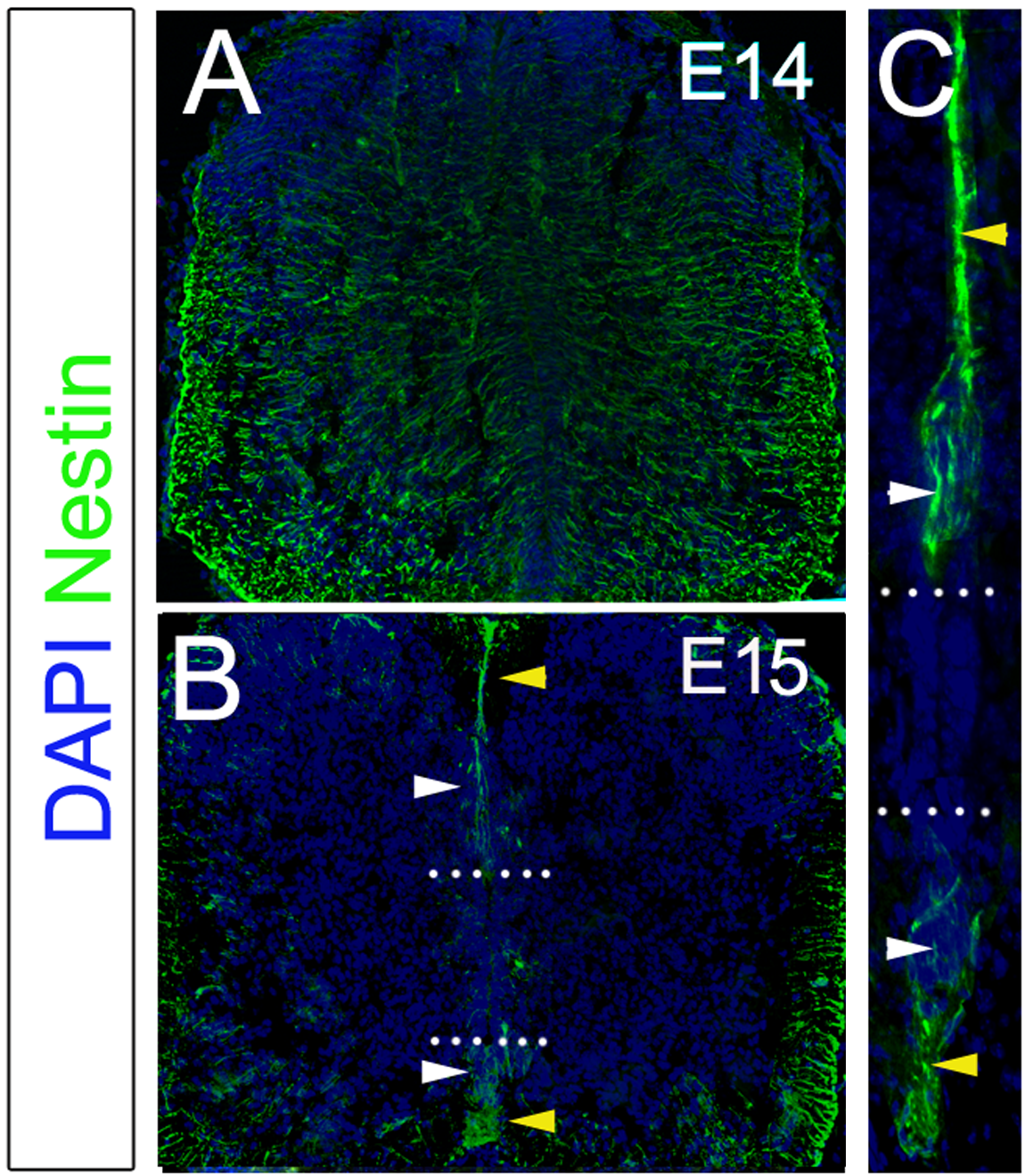

Supplement: S2 Fig — Transverse sections through mouse embryonic spinal cord. (A) At E14 Nestin is detected on mediolateral radial glia. (B, C) By E15, strong Nestin labelling is detected on dorsal and ventral midline radial glial cells (white arrows) that project through the dorsal and ventral funiculi (yellow arrowheads) to the pial surface. Dotted lines demarcate lumen ends. dmNes+RG, dorsal midline Nestin(+) radial glia. (TIF) [file pbio.3000470.s002.tif]

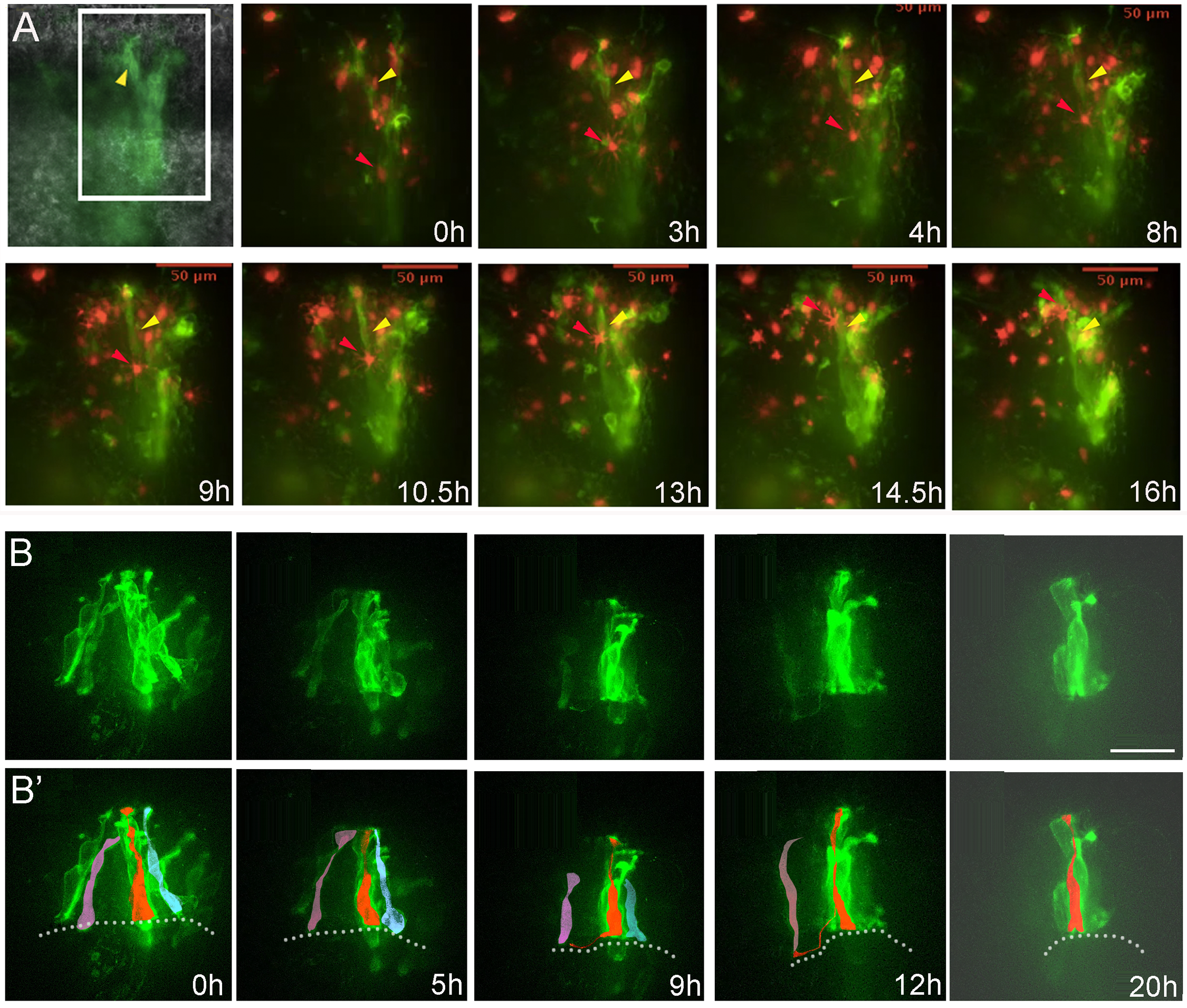

Supplement: S3 Fig — (A) Sequential stills from time-lapse imaging (S2 Movie) after high-density electroporation of membrane-GFP histone-RFP into mouse spinal cord slice. Yellow arrowhead points to a dmNes+RG, whose position remains the same throughout the culture. Red arrowhead points to a nucleus that migrates dorsally. (B) Sequential stills from time-lapse imaging (S4 Movie) after low-density electroporation of membrane-GFP into chick spinal cord slice. (B′) Same images as in (B); cells colour-coded. dmNes+RG cell (red) elongates (9–12 hours) to contact a dVL cell (pink; contact at 9 hours); on the other side, a dVL cell (blue) ratchets up to the dmNes+RG (0–5 hours). dmNes+RG, dorsal midline Nestin(+) radial glia; dVL, dorsal ventricular layer; GFP, green fluorescent protein; RFP, red fluorescent protein. (TIF) [file pbio.3000470.s003.tif]

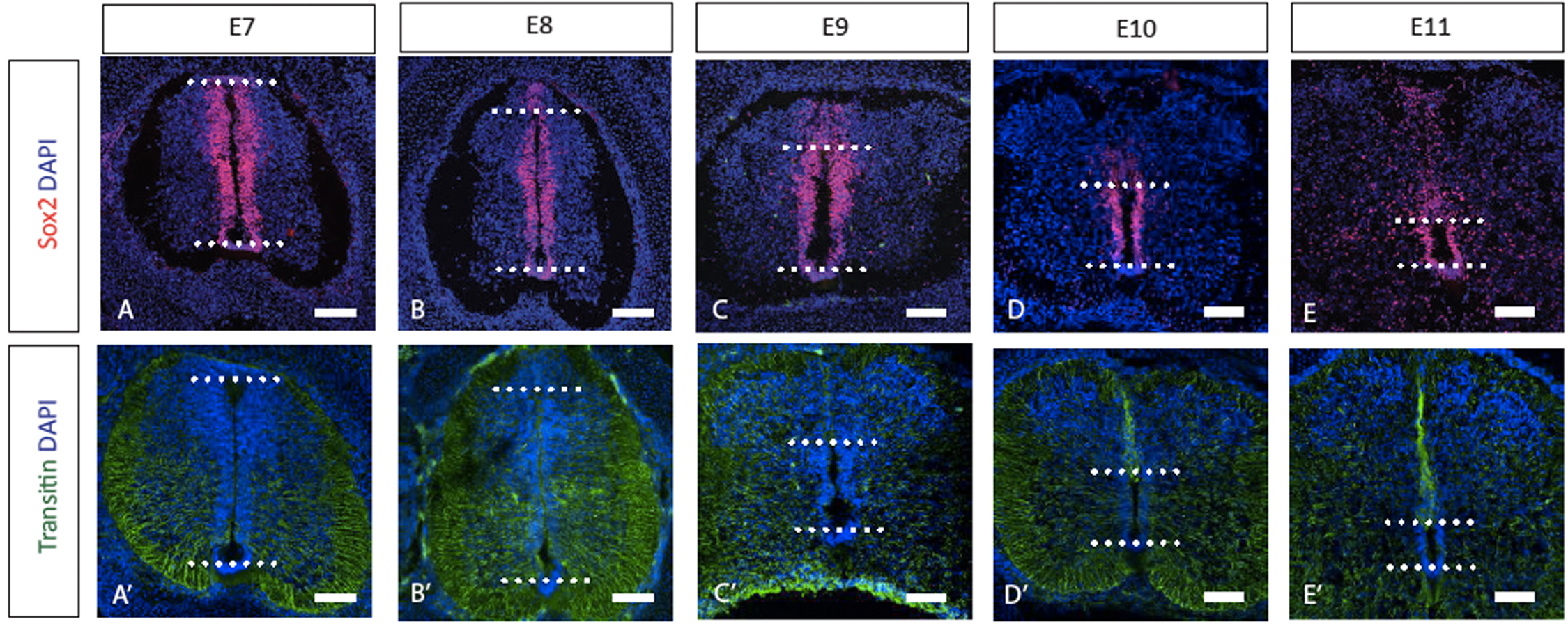

Supplement: S4 Fig — (A, A′–E, E′) Serial adjacent transverse sections through chick embryonic spinal cord between developmental stages E7 and E11. Expression of Sox2/Transitin (a Nestin-like protein) mirrors that of Sox2/Nestin in mouse embryonic spinal cord. As in mouse, dmTransitin+RG stretch from the lumen to the pia. Sox2 cells are found throughout the collapsing VL, as well as dorsal to the obliterated lumen, closely associated with Transitin(+) radial glial processes. dmTransitin+RG, dorsal midline Transitin-expressing radial glia; Sox2, SRY_related HMG-box 2; VL, ventricular layer. (TIF) [file pbio.3000470.s004.tif]

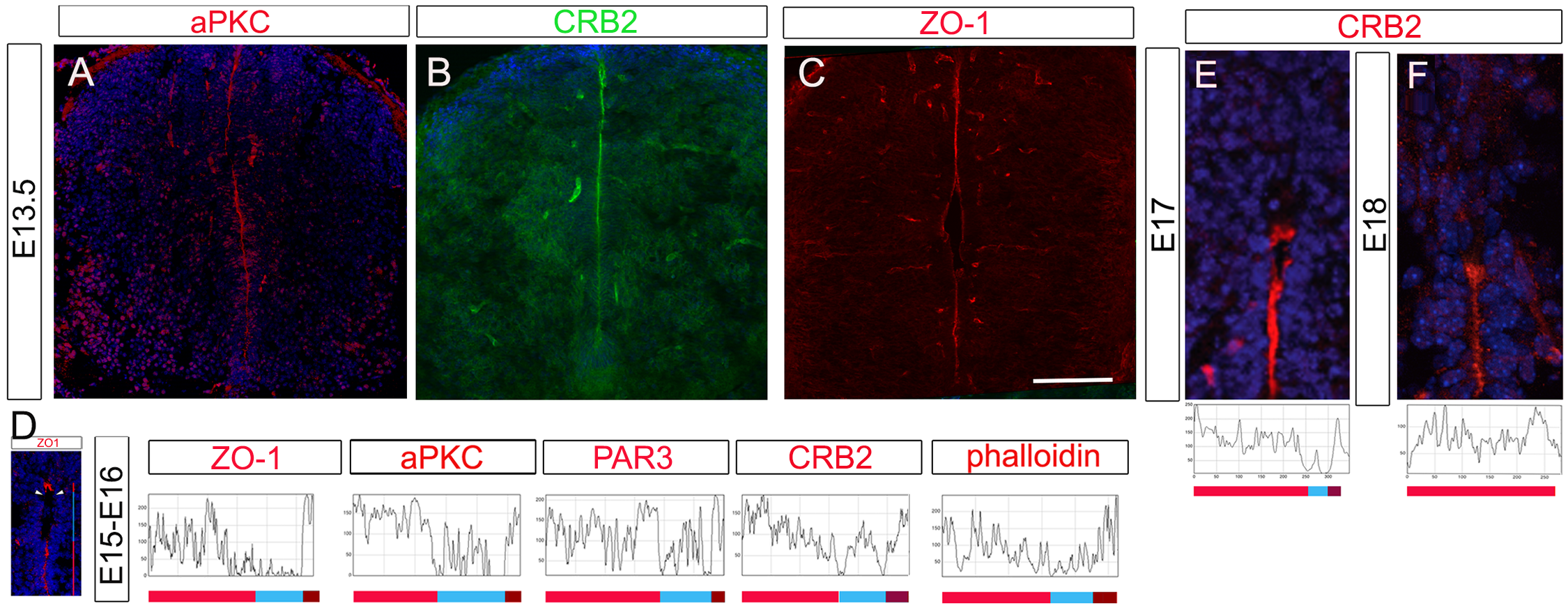

Supplement: S5 Fig — (A-E) Transverse sections through E13.5 (A-C), E17 (E), or E18 (F) mouse spinal cord, analysed by immunohistochemistry as shown. (D) Plots show intensity of labelling along the apical side of VL, from vVL (red) to dVL (blue) to dmNes+RG (dark red) in representative sections analysed at E15.5. dmNes+RG, dorsal midline Nestin(+) radial glia; dVL, dorsal ventricular layer; VL, ventricular layer; vVL, ventral ventricular layer. (TIF) [file pbio.3000470.s005.tif]

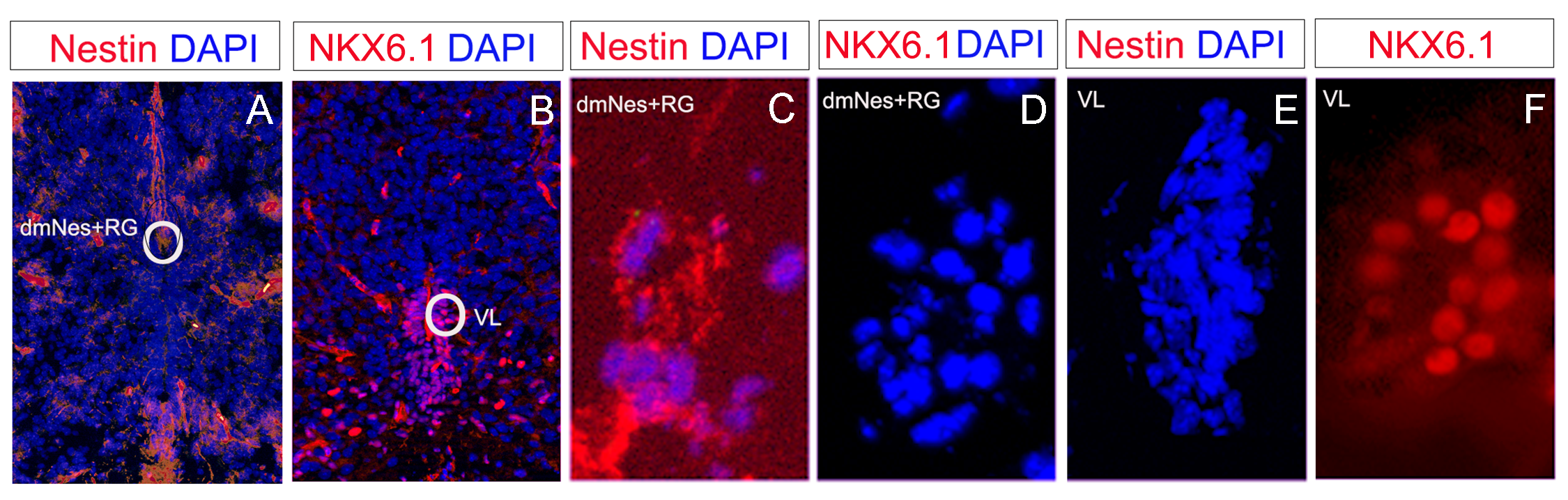

Supplement: S6 Fig — (A, B) Transverse sections, immunolabelled to show position of dmNes+RG (A) or NKX6.1+ vVL cells (B). Circles indicate punched regions. (C-F) Accuracy of punches confirmed through immunolabelling. dmNes+RG express Nestin but not Nkx6.1 (C,D). vVL cells express NKX6.1 but not Nestin (E,F). dmNes+RG, dorsal midline Nestin(+) radial glia; NKX6.1, NK6 homeobox 1; vVL, ventral ventricular layer. (TIF) [file pbio.3000470.s006.tif]

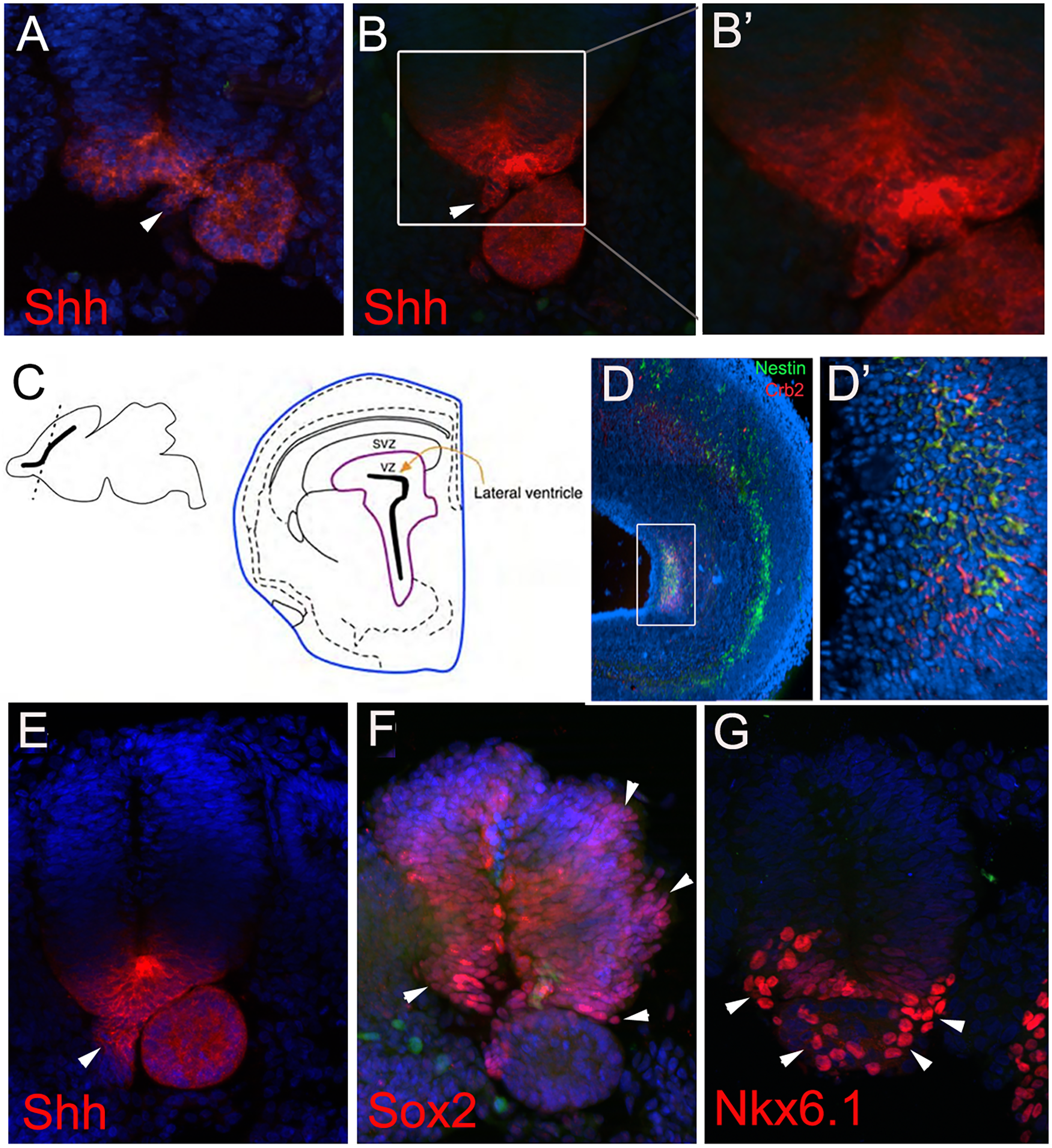

Supplement: S7 Fig — (A-C) Transverse sections through HH st14 chick embryonic neural tube, 24 hours after transplantation with E15.5 dmNes+RG tissue. Shh(+) floor plate cells appear to dissociate. (C) Schematic showing position of SVZ in mouse E17.5 telencephalon. (D) Dorsal SVZ cells co-express Nestin and CRB2; the latter appears non-apical. (D′) High-power view of boxed region. (E-G) Transverse sections through HH st14 chick embryonic neural tube, 24 hours after transplantation with E17.5 mouse SVZ tissue. (E) Shh is detected on cell clumps that appear to have dissociated from the floor plate. (F,G) Sox2 and Nkx6.1 progenitors are located ectopically outside the neural tube. CRB2, Crumbs2; dmNes+RG, dorsal midline Nestin(+) radial glia; Nkx6.1, NK6 homeobox 1; Shh, Sonic hedgehog; Sox2, SRY-related HMG-box 2; SVZ, subventricular zone. (TIF) [file pbio.3000470.s007.tif]

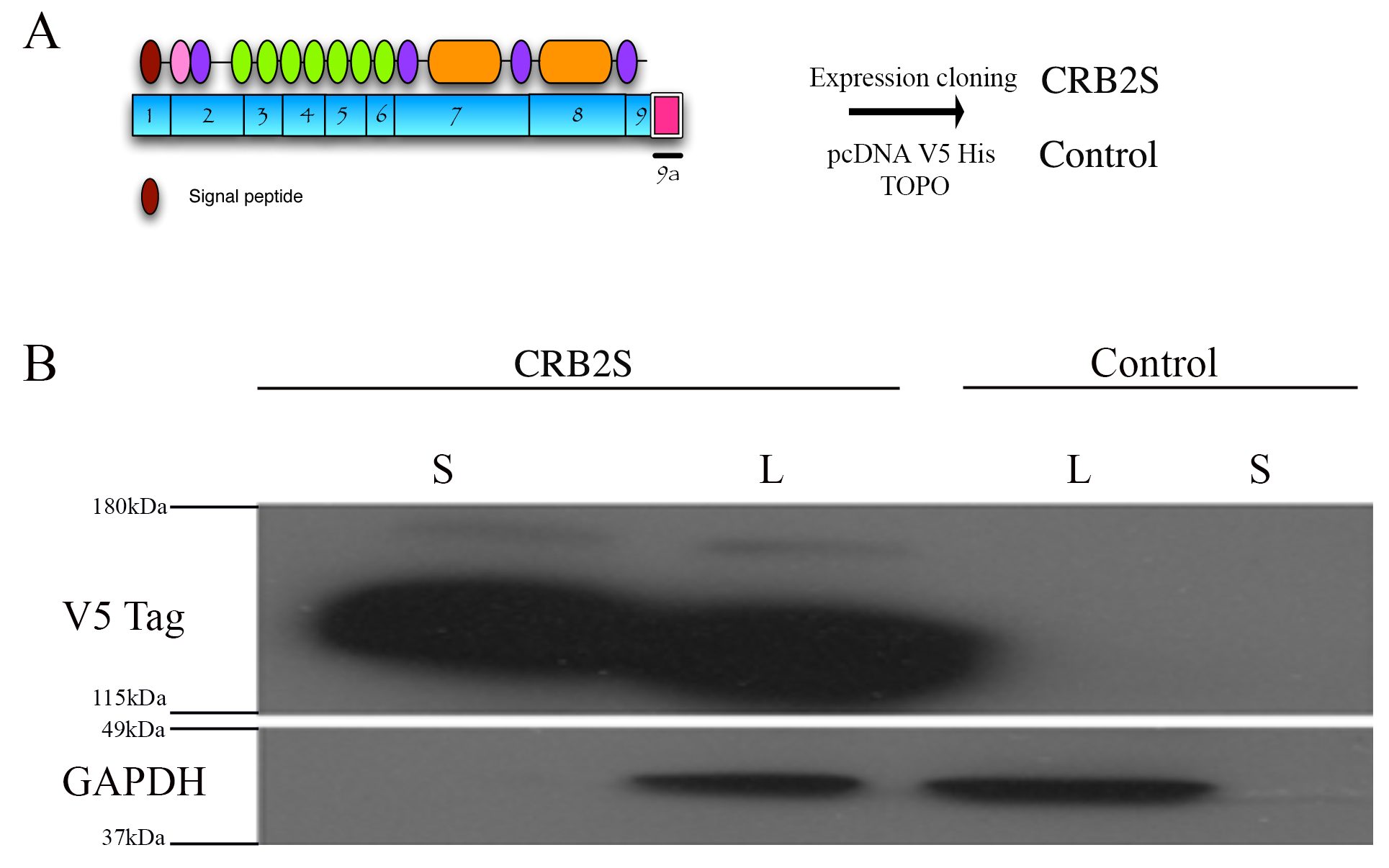

Supplement: S8 Fig — (A) Crb2S cDNA and Crb2S signal peptide coding cDNA were cloned into pcDNA3.1 V5-His-Top expression vector. (B) Western blotting to detect the V5-tagged recombinant protein shows that CRB2S can be detected in the supernatant (S) and lysate (L) from cells transfected with the Crb2S expression vector. Cells were cultured in serum-reduced conditions for 72 hours before harvesting. GAPDH was used as a loading control. Apparent molecular weights are indicated on the left in B. CRB2S, secreted CRB2; GAPDH, glyceraldehyde 3-phosphate dehydrogenase. (TIF) [file pbio.3000470.s008.tif]

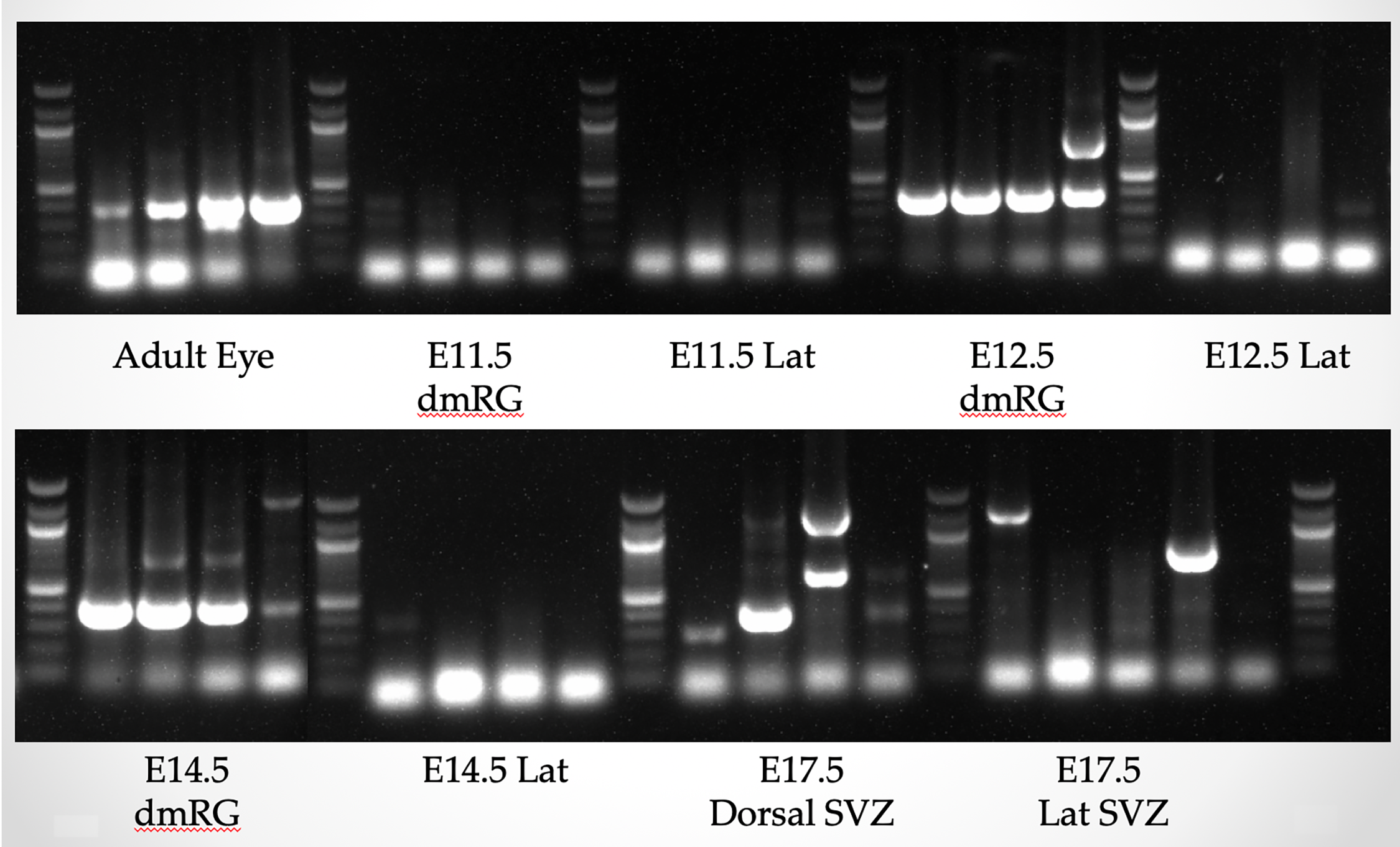

Supplement: S9 Fig — dmNes+RG, dorsal midline Nestin(+) radial glia; SVZ, subventricular zone; VL, ventricular layer. (TIF) [file pbio.3000470.s009.tif]

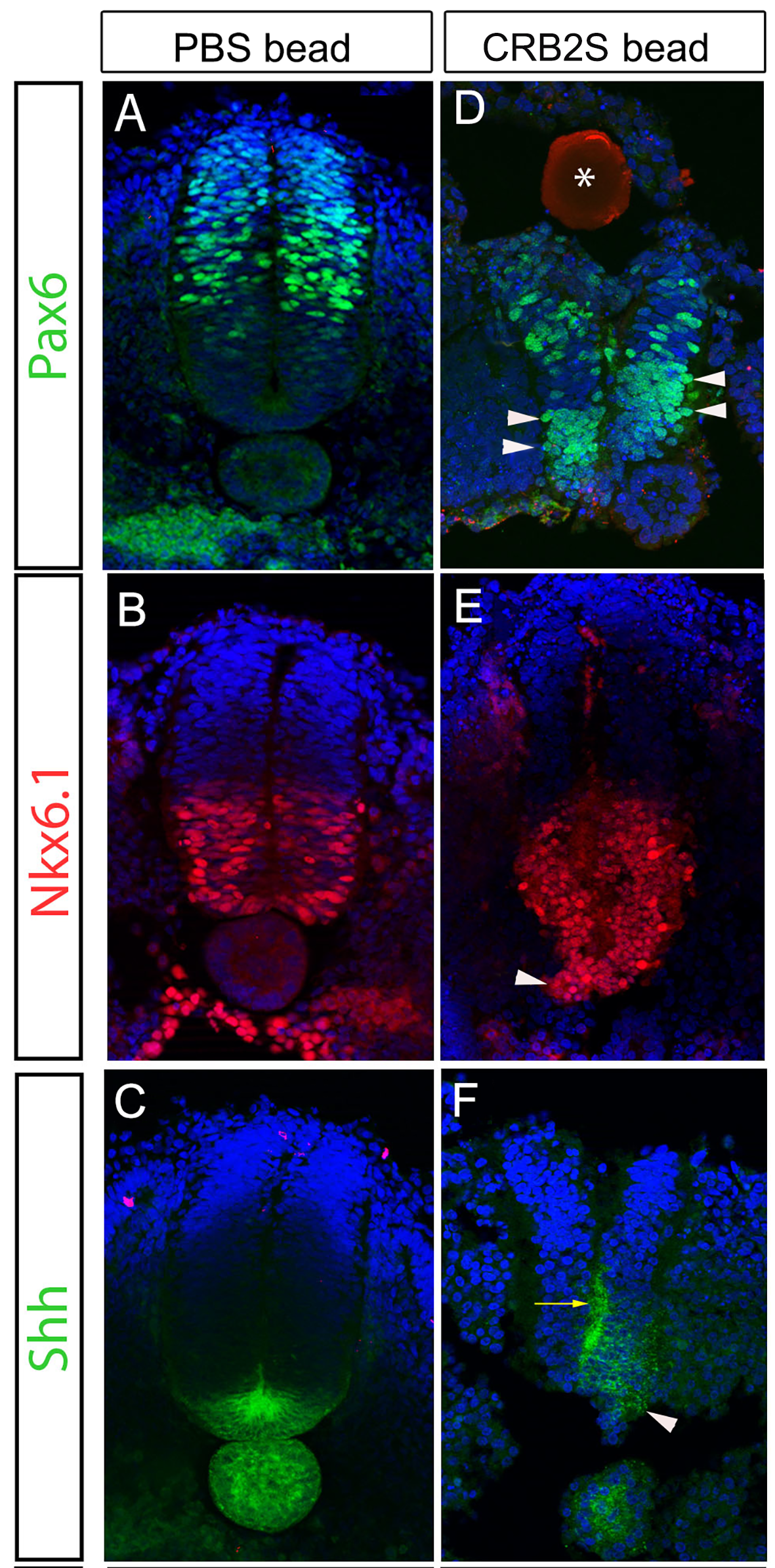

Supplement: S10 Fig — Transverse serial adjacent sections through HH st14 chick embryonic neural tubes, 24 hours after implantation of PBS-soaked (A-C) or CRB2S-soaked (D-F) beads. (A-C) PBS-soaked beads do not disrupt Pax6(+) dorsal progenitors, Nkx6.1(+) ventral progenitors, or Shh(+) floor plate cells. (D-F) CRB2S-soaked beads caused delamination of neural tube progenitors: Pax6(+) and Nkx6.1(+) progenitors are mislocalised/mispatterned and detected outside of the neural tube (arrowheads). Shh expands dorsally and is detected on cell clumps that appear to have pinched off from the floor plate. Asterisk in (D) points to bead. Underlying data shown in S5 Table. CRB2S, secreted CRB2; Nkx6.1, NK6 homeobox 1; Pax6, paired-box 6; Shh, Sonic hedgehog; Sox2, SRY-related HMG-box 2. (TIF) [file pbio.3000470.s010.tif]

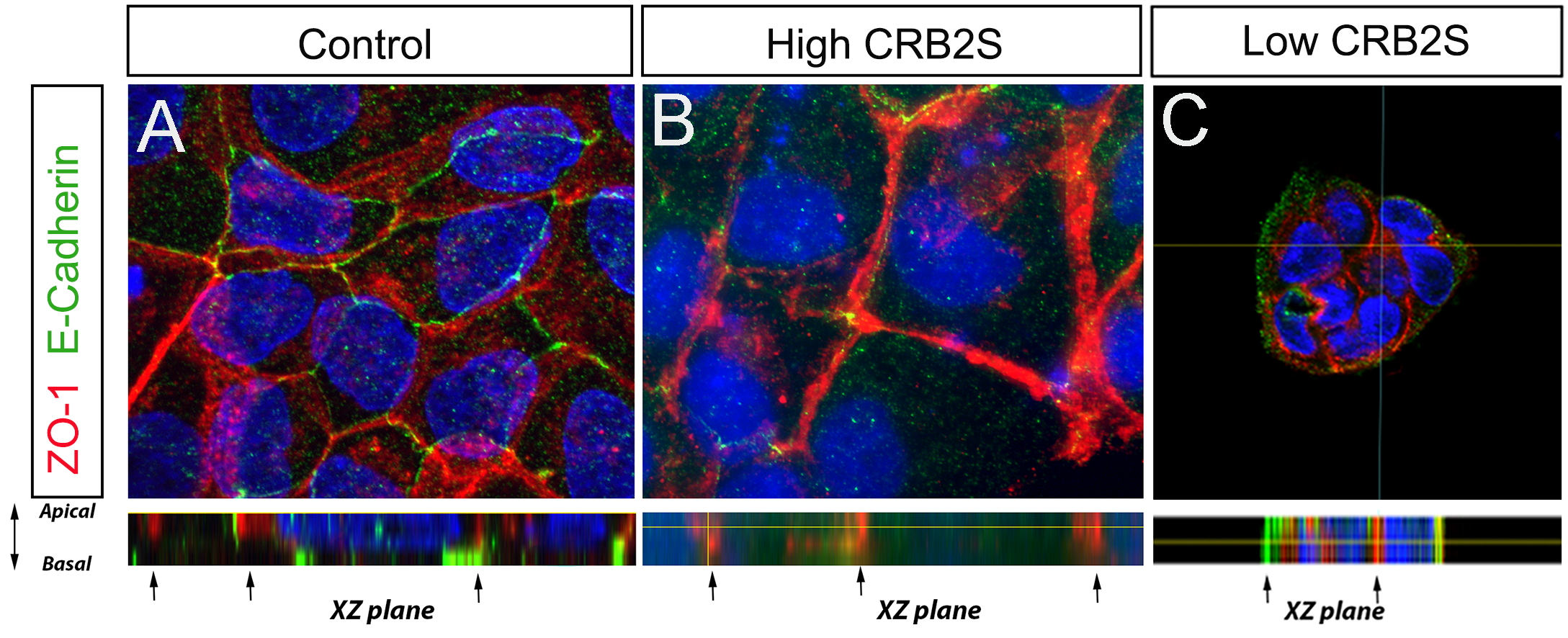

Supplement: S11 Fig — MDCK cells, cultured at high density in control medium (A) or a high concentration of CRB2S (B) or at low density with a low concentration of CRB2S (C), immunolabelled with Zo-1 and E-cadherin. XZ-plane views show a disruption in polarity in the presence of CRB2S. Underlying data shown in S6 Table. CRB2S, secreted CRB2; ZO-1, Zona occludens 1. (TIF) [file pbio.3000470.s011.tif]

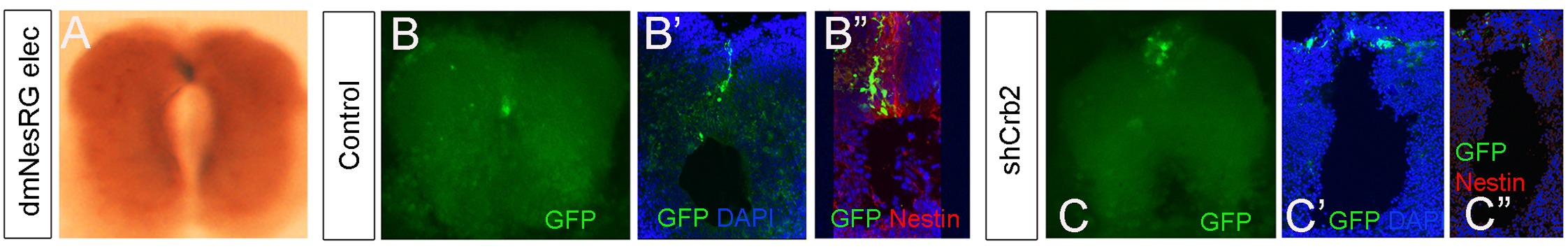

Supplement: S12 Fig — (A) At 0 hours, fast-green shows targeted electroporation to roof plate/dmNes+RG. (B-B″) After a 72-hour culture, slices targeted with a control GFP construct showed a 4-fold collapse, i.e., similar to that in vivo. Analysis of whole-mount slices showed GFP at the dorsal lumen (B), and analysis of sections revealed GFP in elongated dmNes+RG (B′,B″). (C-C″) By contrast, after targeting dmNes+RG with shCrb2, no collapse is detected (C) and no elongated dmNes+RG can be detected (C′,C″). dmNes+RG, dorsal midline Nestin(+) radial glia; GFP, green fluorescent protein. (TIF) [file pbio.3000470.s012.tif]
