# Supplementary material for: Crumbs2 mediates ventricular layer remodelling to form the spinal cord central canal
Source: PLoS Biol. 2020 Mar 9;18(3):e3000470. doi: 10.1371/journal.pbio.3000470 (PMC7108746; doi:10.1371/journal.pbio.3000470)
Supplement: S1 Table — Three embryos were analysed at each stage; each row shows measurement from one 15-μm section. VL, ventricular layer. (DOCX) [file pbio.3000470.s013.docx]

|  | **E14** | **E15** | **E16** | **E17** |
| --- | --- | --- | --- | --- |
| **Embryo 1** | 505 | 196 | 140 | 115 |
|  | 488 | 188 | 125 | 116 |
|  | 464 | 162 | 107 | 108 |
| **Embryo 2** | 520 | 238 | 109 | 97 |
|  | 502 | 217 | 98 | 106 |
|  | 492 | 205 | 104 | 102 |
| **Embryo 3** | 515 | 212 | 130 | 105 |
|  | 510 | 219 | 122 | 103 |
|  | 487 | 176 | 125 | 97 |
| **Mean** | **498.11** | **201.44** | **117.78** | **105.44** |
| **SEM** | **5.77** | **7.84** | **4.62** | **2.27** |
