# Supplementary material for: Crumbs2 mediates ventricular layer remodelling to form the spinal cord central canal
Source: PLoS Biol. 2020 Mar 9;18(3):e3000470. doi: 10.1371/journal.pbio.3000470 (PMC7108746; doi:10.1371/journal.pbio.3000470)
Supplement: S2 Table — Three embryos were analysed from each stage; each row shows measurement from one 15-μm section. Unpaired Student t test shows significant differences in length of dVL on each consecutive day but no significant difference in length of vVL on consecutive days. ***p < 0.001; **p = 0.0011. dVL, dorsal ventricular layer; vVL, ventral ventricular layer. (DOCX) [file pbio.3000470.s014.docx]

|  | **E14 dorsal** | **E15 dorsal** | **E16 dorsal** | **E17 dorsal** | **E14 ventral** | **E15**  **ventral** | **E16**  **ventral** | **E17**  **ventral** |
| --- | --- | --- | --- | --- | --- | --- | --- | --- |
| **Embryo 1** | 405 | 104 | 42 | 0 | 100 | 92 | 98 | 115 |
|  | 342 | 111 | 25 | 11 | 146 | 77 | 100 | 105 |
|  | 334 | 60 | 12 | 10 | 130 | 102 | 95 | 98 |
| **Embryo 2** | 426 | 105 | 10 | 0 | 94 | 133 | 89 | 97 |
|  | 418 | 117 | 13 | 11 | 84 | 100 | 85 | 95 |
|  | 376 | 110 | 19 | 7 | 116 | 95 | 85 | 95 |
| **Embryo 3** | 410 | 90 | 25 | 10 | 105 | 122 | 105 | 95 |
|  | 410 | 114 | 30 | 13 | 100 | 105 | 92 | 90 |
|  | 387 | 70 | 35 | 7 | 100 | 106 | 90 | 90 |
| **Mean** | **389.78** | **96.78** | **23.44** | **7.67** | **108.33** | **104.67** | **93.22** | **97.78** |
| **SEM** | **11.02** | **6.72** | **3.66** | **1.58** | **6.42** | **6.26** | **2.28** | **2.62** |

***** *** ****
