# Supplementary material for: Crumbs2 mediates ventricular layer remodelling to form the spinal cord central canal
Source: PLoS Biol. 2020 Mar 9;18(3):e3000470. doi: 10.1371/journal.pbio.3000470 (PMC7108746; doi:10.1371/journal.pbio.3000470)
Supplement: S3 Table — Between E14 and E17, there is a large and similar proportional reduction of SOX2(+) and PAX6(+) nuclei (83.6% and 86.9%, respectively), but only a small reduction (23.5%) of NKX6.1(+) nuclei. Nkx6.1, NK6 homeobox 1; PAX6, paired-box 6; SOX2, SRY-related HMG-box 2; VL, ventricular layer. (DOCX) [file pbio.3000470.s015.docx]

|  | **E14** | **E15** | **E16** | **E17** |
| --- | --- | --- | --- | --- |
| **Sox 2** | 495, 520 | 261, 278 | 119, 108 | 82, 76 |
|  | 508, 522 | 230, 225 | 106, 102 | 70, 77 |
|  | 488, 472 | 300, 260 | 90, 113 | 89, 101 |
| **Nkx6.1** | 83, 77 | 72, 81 | 67, 72 | 68, 64 |
|  | 91, 76 | 71, 67 | 75, 60 | 58, 70 |
|  | 80, 88 | 60, 79 | 59, 70 | 64, 57 |
| **Pax6** | 117, 121 | 35, 40 | 38, 42 | 16, 14 |
|  | 127, 131 | 38, 41 | 37, 35 | 20, 12 |
|  | 105, 101 | 50, 45 | 32, 34 | 13, 17 |
