# Supplementary material for: Crumbs2 mediates ventricular layer remodelling to form the spinal cord central canal
Source: PLoS Biol. 2020 Mar 9;18(3):e3000470. doi: 10.1371/journal.pbio.3000470 (PMC7108746; doi:10.1371/journal.pbio.3000470)
Supplement: S4 Table — Analyses based on measurements from 2 embryos, 2 sides. dVL, dorsal ventricular layer; vVL, ventral ventricular layer. (DOCX) [file pbio.3000470.s016.docx]

|  | **Dorsal VL** | **Ventral VL** |
| --- | --- | --- |
| **Embryo 1, side 1** | 24 | 56 |
| **Embryo 1, side 2** | 22 | 58 |
| **Embryo 2, side 1** | 21 | 52 |
| **Embryo 2, side 2** | 19 | 50 |
