# Supplementary material for: Crumbs2 mediates ventricular layer remodelling to form the spinal cord central canal
Source: PLoS Biol. 2020 Mar 9;18(3):e3000470. doi: 10.1371/journal.pbio.3000470 (PMC7108746; doi:10.1371/journal.pbio.3000470)
Supplement: S5 Table — Number of embryos showing strong, subtle, or no phenotype after transplantation of dmNes+RG, VL cells, ventral radial glia (RG), CRB2S-soaked beads, PBS-soaked beads, or SVZ cells. CRB2S, secreted CRB2; dmNes+RG, dorsal midline Nestin(+) radial glia; SVZ, subventricular zone; VL, ventricular layer. (DOCX) [file pbio.3000470.s017.docx]

| **Transplantation**  **type** | **No. of embryos analysed** | **No. of embryos showing a strong phenotype or (subtle phenotype) for** | | | | | | | | | **No. embryos with no phenotype** |
| --- | --- | --- | --- | --- | --- | --- | --- | --- | --- | --- | --- |
|  |  | **Laminin** | **D/glycan** | **Sox2** | **Shh** | **Pax6** | **Nkx6.1** | **aPKC** | **Zo-1** | **CRB2** |  |
| **VL cells** | 8 (24h)  4 (20h) | 0 (1) | 0 (2) | 0 | 0 | 0 | 0 | 0 | 0 | 0 | 10 |
| **dm Nes+ RG** | 11 (24h)  4 (20h) | 6 (2) | 5 (3) | 6 (2) | 6 (2) | 5 (3) | 4 (4) | 3 | 3 | 3 | 3  1 |
| **Ventral RG cells** | 3 | 0 | 0 | 0 | 0 | 0 | 0 | ND | ND | ND | 3 |
| **CRB2S soaked beads** | 7  4 | 4 (2) | 4 (2) | 4 (2) | 4 (2) | 4 (2) | 4 (2) | 3 | 2 (1) | 2 | 1  1 |
| **PBS soaked bead** | 6  4 | 0 (2) | 0 (2) | 0 | 0 | 0 | 0 | 0 | 0 | 0 | 4  4 |
| **SVZ cells** | 5  1 | 5 | 5 | 5 | 5 | 5 | 5 | ND | 1 | ND | 0  0 |
| **Other (lateral tissue from neural plate, midbrain, hypothalamus)** | 3 each | 0 (2) | 0 (1) | 0 | 0 | 0 | 0 | 0 | 0 | 0 | 6 |
