# Supplementary material for: Crumbs2 mediates ventricular layer remodelling to form the spinal cord central canal
Source: PLoS Biol. 2020 Mar 9;18(3):e3000470. doi: 10.1371/journal.pbio.3000470 (PMC7108746; doi:10.1371/journal.pbio.3000470)
Supplement: S6 Table — After culture, cells were immunolabelled to detect ZO-1 and E-cadherin. Five random fields were selected (n = 2 experiments) and XZ-plane views analysed for 20 cells per field. Cells were scored as polarised if ZO-1 was detected apically. Table shows number cells/field showing apical ZO-1; mean values and SEM shown in bottom line. CRB2S, secreted CRB2; ZO-1, Zona occludens 1. (DOCX) [file pbio.3000470.s018.docx]

|  | **Control Medium** | **CRB2S** |
| --- | --- | --- |
| **Field 1** | 16/20 | 4/20 |
| **Field 2** | 17/20 | 5/20 |
| **Field 3** | 14/20 | 3/20 |
| **Field 4** | 15/20 | 6/20 |
| **Field 5** | 15/20 | 5/20 |
|  | **15.4 +/- 0.5 (sem)** | **4.6 +/- 0.5 (sem)** |
