# Supplementary material for: Crumbs2 mediates ventricular layer remodelling to form the spinal cord central canal
Source: PLoS Biol. 2020 Mar 9;18(3):e3000470. doi: 10.1371/journal.pbio.3000470 (PMC7108746; doi:10.1371/journal.pbio.3000470)
Supplement: S7 Table — Five random wells were selected and cells counted at 0 hours or after a 15-hour culture in control medium, CRB2S (low concentration), and CRB2S (high concentration). Bottom line shows mean values and SD. CRB2S, secreted CRB2. (DOCX) [file pbio.3000470.s019.docx]

|  | **0hrs** | **15hrs Control** | **15hrs Crb2S (low)** | **15hrs Crb2S (high)** |
| --- | --- | --- | --- | --- |
| **Well 1** | 39 | 66 | 55 | 60 |
| **Well 2** | 30 | 52 | 67 | 50 |
| **Well 3** | 26 | 64 | 63 | 45 |
| **Well 4** | 29 | 55 | 65 | 56 |
| **Well 5** | 35 | 54 | 60 | 51 |
|  | **31.8+/- 5.2 (SD)** | **58.2+/-6.3 (SD)** | **62.0+/-4.7 (SD)** | **52.4+/-5.8 (SD)** |
