# Supplementary material for: Crumbs2 mediates ventricular layer remodelling to form the spinal cord central canal
Source: PLoS Biol. 2020 Mar 9;18(3):e3000470. doi: 10.1371/journal.pbio.3000470 (PMC7108746; doi:10.1371/journal.pbio.3000470)
Supplement: S8 Table — Three random fields were selected (from 3 random wells) and a total of 15 clumps counted after a 15-hour culture in control medium, CRB2S (low concentration), and CRB2S (high concentration). Bottom line shows mean values and SEM. CRB2S, secreted CRB2. (DOCX) [file pbio.3000470.s020.docx]

| **Clump** | **Control** | **Crb2S (low)** | **Crb2S (high)** |
| --- | --- | --- | --- |
| **1** | 5 | 3 | 2 |
| **2** | 4 | 7 | 2 |
| **3** | 3 | 10 | 3 |
| **4** | 7 | 3 | 2 |
| **5** | 10 | 4 | 1 |
| **6** | 6 | 4 | 2 |
| **7** | 7 | 5 | 2 |
| **8** | 4 | 2 | 2 |
| **9** | 5 | 2 | 2 |
| **10** | 6 | 3 | 2 |
| **11** | 3 | 4 | 2 |
| **12** | 5 | 3 | 1 |
| **13** | 5 | 2 | 2 |
| **14** | 4 | 5 | 2 |
| **15** | 4 | 6 | 2 |
|  | **5.2+/-0.5 (sem)** | **4.2+/-0.6 (sem)** | **2.0 +/-0.1 (sem)** |
