# Supplementary material for: Crumbs2 mediates ventricular layer remodelling to form the spinal cord central canal
Source: PLoS Biol. 2020 Mar 9;18(3):e3000470. doi: 10.1371/journal.pbio.3000470 (PMC7108746; doi:10.1371/journal.pbio.3000470)
Supplement: Attachment — Submitted filename: CTait Rev responses 06012020.docx [file pbio.3000470.s027.docx]

We thank both Reviewers for the time they have taken to look through our data and suggest improvements. Both made really useful and helpful points. The two key points, made by each in different ways, were that we needed to (1) strengthen the evidence for a specific delamination mechanism, and (2) strengthen the evidence for a role for CRB2S in dmNes+RG.

**For (1) we have performed new in vivo imaging studies and re-analysed our initial data using new image deconvolution software. Our new data provides much better cell resolution than previously, and shows the ratcheting of the dmNes+RG endfoot that accompanies delamination. The new imaging studies also reveal cell interactions and behaviours that we could not detect previously, indicative of a specific delamination mechanism. The new data is shown in Movies 1 (which is the original Movie, but with the new deconvolution software) and Movies 2-6 (which show new data). Still images from the new data are shown in Figure 3 and Supp. Fig.3B.**

**For (2) we have developed a new *ex vivo* slice culture, where dmNes+RG are specifically targeted, either through implantation of beads soaked with anti-CRB2 antibody or *Crb2* knockdown (using an shRNA construct). In both assays, collapse is prevented. The new data is shown in Figure 7O-U and new Supp. Fig.11.**

**In addition, we have performed additional analyses and edits as suggested by each Reviewer. Our detailed responses are described below:**

Reviewer #1: This manuscript describes a detailed analysis of the process of dorsal collapse in the mouse spinal cord and presents evidence that the dorsal midline radial glia trigger this collapse by producing a secreted form of Crumbs2 that induces the adjacent ventricular layer cells to lose polarity and delaminate. This is a very interesting set of results that tell an important and unexpected story, making it eminently suitable for PLoS Biology. However, there are several ways that the manuscript could be improved as outlined below:

1)  As a non-mouse, non-neural developmental biologist, I found the initial description of the cell-types involved in dorsal collapse very hard to follow. It would help a lot to have a well-labelled diagram in figure 1

**We thank the reviewer for pointing out a way to make the data more accessible: we have added a labelled schematic (Figure 1P).**

2) The EM images in figure 4 are difficult to interpret as the boundaries between cells are not clear. This makes it a matter of trust for the reader to believe that the green arrows mark junctions between cells. This is complicated by the fact that the green arrows only mark the junctions that are not apparent and the prominent junctions are not labelled.

**We have altered Figure 4 to better show sequential junctions down one side of the lumen: the new figure shows the transition from long and prominent junctions (dorsally) to, short, non-prominent junctions (on delaminating VL cells), to lengthening junctions more ventrally. Additionally, we clarify that the junctions appear to match cell boundaries.**

3a) The authors argue that Crb2S disrupts epithelial polarity in the ventricular layer, which leads to delamination, but it is not clear from their data that this is the correct causal connection. In figure 4, ZO-1 and aPKC disappear from a number of VL cells, not just those that are delaminating.

**When we first started analysing these experiments we thought this was the case – ie that Zo-1 and aPKC disappear from cells that will (but have not yet) delaminated. However, on close inspection we realised that although these markers are reduced they are only completely undetectable on the delaminating VL cells that are immediately adjacent to the Nes+RG cell. We have increased the contrast on the figures to make this more clear and show the data quantitatively in Supp. Fig.3. We therefore stand by the assertion that loss (failure to detect) is restricted to delaminating cells. We do agree, though, that the action of Crb2/Crb2S in dorsal collapse is likely to be part of a more complex system: the looser arrangement of dorsal VL cells around a wider lumen suggests that Crb2S may operate on cells that are already sensitised. We clarify this in the Discussion. Nonetheless, our gain of function studies (Figure 5) indicate a causal connection.**

3b) In addition, the cells appear to delaminate by apical abscission, which is a completely different mechanism to a loss of the apical domain.

**In response to this comment, and Reviewer 2 we have re-analysed our mouse images using new deconvolution software and performed additional live imaging. The new data is shown in Movies 1, 2-6, new Figure 3 and Supp. Fig.3B. In addition to analysing new mouse cultures, we have performed new imaging studies in chick spinal cord slices, where cell behaviours can be more clearly discerned. Based on our new analyses we no longer use the term abscission: a thin cellular thread (that we could not see previously) seems to persist before the entire cell delaminates (eg see Movie 3, 8 seconds). Analysis of mouse spinal cord slices suggests the same (Movie 6). We therefore now refer to delamination, but not to apical abscission.**

4) The effects of Crb2S on MDCK cells would be more compelling if the untreated cells were polarised, but this is hard to see in the images shown.

**We now provide xz views, showing Zo-1 at the apical domain and E-Cad in the basal domain in control cells, and their disruption in cells treated with a medium concentration of Crb2S. The new data is shown in (new) Supp. Fig.10.**

5a)  The Crb2 knockout in the Nestin +ve cells gives a very convincing phenotype, but left me confused. Is the Cre recombinase expressed only in the dmNes+RG cells, which are the Nestin positive cells visible in figure 2, or is it expressed in the whole ventricular layer as stated in the text?  If the latter is the case, this does not prove a role for Crb2S in delamination as the effect could be due to the loss of the transmembrane form in the VL cells.

**We apologise for the lack of clarity. The second interpretation is correct, ie the text and title of the paper (‘A role for Crb2’), as this experiment shows only that the transmembrane form of Crb2 is needed on VL cells. We did not mean to suggest otherwise, and included Supp.Fig.2 to show that prior to E13, Nestin is detected on VL cells. We have made a number of changes to the wording of the manuscript to clarify this point. The key point is that if VL cells do not have the transmembrane form of Crb2, then collapse does not occur.**

**The reviewer is absolutely correct that this experiment does not prove a role for Crb2S in delamination. Importantly we have now conducted two new sets of experiments, targeting Crb2 specifically in the dmNes+RG. cells. These experiments show that collapse does not occur when *Crb2* is knocked out by shRNA or the protein blocked using a bead-soaked anti-Crb2 antibody. The latter treatment results in the loss of Crb2S. The new data are shown in Fig.7 and Supp. Fig.11.**

5b) However, this would also show that loss of apical Crb2 and aPKC from the VL cells does not lead to a loss of polarity or delamination. Please clarify.

**Our data suggests that the interaction of Crb2S with Crb2 leads to specific changes in cell polarity that initiate cell delamination. For instance, we see a downregulation of PAR3 in vivo in delaminating cells, but not after genetic loss of Crb2. We now clarify these points in the Discussion.**

6) The authors propose in the discussion that loss of Crb2 and aPKC promotes the formation of an apical actin cable in the VL cells, but this does not seem consistent with their data. The dorsal VL cells with reduced Crb2 and aPKC appear to have less apical F-actin than other cells, and this does not form the straight line that one would expect for a supracellular actin able.

**We thank the reviewer for this comment, and now do not propose this.**

Reviewer #2: This paper investigates the mechanism of ‘dorsal collapse’ whereby the originally extensive neural tube lumen is converted through development to a much smaller, ventrally located lumen. Concomitantly, the ependymal cell population becomes defined. This phenomenon is well described and is known to involve transformation of radial glia and a key role of apical proteins in the neuroepithelium. The authors investigate the mechanism further in mouse and chick, and obtain evidence for a non-cell-autonomous role of a secreted form of the Crumbs2 protein.

The paper is interesting and the studies have been carefully performed. The work is well described and documented in the main and supplementary figures. The paper divides into two parts. The first is a molecular anatomical study, in which a key role for the nestin-positive radial glia is mainly studied. I would say this is valuable in extending existing knowledge on dorsal collapse. The second part is experimental, involving testing of the hypothesis that the CRB2S secreted variant plays an essential role in dorsal collapse. This part of the study is innovative and has the potential to significantly advance this research field. However, I feel the experimental studies currently fall short of providing convincing evidence to support the CRB2S hypothesis.

1) Ratcheting is proposed as a mechanism of dorsal collapse. Live imaging of sparsely transfected spinal cord slice cultures provides evidence for this. In Fig 3A, I can clearly see the second dorsal VL progenitor cell making contact with the dmNes+RG cell, in frames 990-1060, after apical abscission of the previous contacting cell. Interestingly, Movie 1 seems to stop before this critical event - is it missing the last frames? In any case, how do the authors know that after abscission of a dorsal VL progenitor, there is “ratcheting down” so that the next one (ventrally) then makes contact? I cannot really see convincing evidence for this conclusion. For example, is there a movie in which a labelled (and so clearly more ventral) VL cell becomes connected after a clearly more dorsal one has abscised?

**We thank the reviewer for this comment, which has prompted us to improve and expand on these experiments in 2 ways:**

**First we have re-analysed the original live-images with new deconvolution software, and now present the data as a new figure (Figure 3A,A’ and Movie 1). In many of the live imaging studies, electroporated cells move in and out of focus (which is why the reviewer asked whether the movie was missing the last frames). In the new Movie, VL cells can be seen that stay in focus throughout, and we demonstrate progressive delamination.**

**Nonetheless, even with the much-improved deconvolution software we agree that it is difficult to discern individual cell behaviours and cell interactions in the example shown, including the ratcheting down of the dmNes+RG cell. This is partly because, as the dmNes+RG cell lengthens, it thins to the point that it can barely be detected, and partly because the mouse spinal cord cultures are so small, making it difficult to target small numbers of cells. We therefore performed additional live-imaging, using chick spinal cord cultures: these are slightly larger than mouse and it is possible to target fewer cells. We are excited about the new data, which demonstrates more clearly the ratcheting up of dVL cells, their delamination and the concomitant ratcheting down of the dmNes+RG endfoot. The new data is shown in Movies 2-6; Fig.3B,B’,C,C’ and Supp. Fig.3B). Additionally our new data illustrates an interaction that we did not pick up previously – potentially a cellular remodelling event that enables delamination. We describe this in the text.**

2a) The main effect of dmNes+RG cell transplantation into the chick neural tube lumen seems to be breakage of the basement membrane, with cells becoming located outside the normal confines of the spinal cord. A similar effect is observed after implantation of beads soaked in purified CRB2S. However, this basement membrane breakage is not normally observed during dorsal collapse. Perhaps the authors would say their manipulations deliver abnormally large amounts of CRB2S, that have additional effects in the spinal cord?

**We believe that the breakage of the basement membrane is a secondary effect: the first change that we detect is a downregulation of apical and junctional proteins. We now clarify this in the text and have altered the order and labelling of the panels, to help make this point.**

2b) However, I am struggling to see much resemblance between the normal dorsal collapse process and the changes induced by delivering CRB2S. Can the effect be titrated, by implanting beads soaked in smaller amounts of CRB2S? Alternatively, perhaps this gain-of-function experiment is problematic, as the recipient spinal cord is already ‘dorsally collapsing’ and so the additional effects of CRB2S release are not easy to interpret.

**The reviewer is correct in stating that this gain-of-function experiment does not resemble dorsal collapse: the experiment is performed many days before dorsal collapse is initiated, at a time when the neural tube is a neuroepithelium. The key point was to illustrate that excess Crb2S phenocopies the dmNes+RG cell, in terms of its ability to promote early changes (loss of apical and junction proteins) and even to initiate a delamination. As explained in (2a) we have altered the order of the panels and the text, and hope that this point is now more clear.**

2c) Could the authors introduce a blocking antibody to CRB2S that would serve as a loss-of-function experiment, to see if dorsal collapse is diminished?

**We thank the reviewer for encouraging us to try this difficult experiment (difficult because it is impossible to target the dmNes+RG in vivo – even in chick - at the necessary time (E8)). We have therefore worked up an ex vivo slice culture assay, similar to that for live imaging, but cultured for a longer period. We now show dorsal collapse in slice cultures of isolated mouse spinal cord. We demonstrate that a bead soaked in anti-CRB2 Ab, positioned adjacent to the roof plate prevents collapse whereas a control bead does not interfere with collapse. The new data is shown in Figure 7O-U. We were surprised (but pleased) to observe that although we can no longer detect the weak diffuse CRB2 labelling on Nes+RG cells, we can still detect apical CRB2. On this basis, we conclude that the antibody specifically blocks CRB2S in this experiment. (Please note that this is the best experiment that we can perform along these lines: we have tried, for many years, to make an antibody that specifically recognises the Crb2S variant, but have not been successful).**

3. The mouse knockout of Crb2 does indeed produce faulty dorsal collapse, consistent with the authors’ hypothesis. However, one could argue that this is happening because of the need for Crb2 for the vital regulation of apico-basal polarity in the neuroepithelium, and not specifically through interference with the postulated CRB2S mechanism. An obvious further experiment is to genetically prevent CRB2S production, perhaps by introducing a specific mutation to prevent the causative splicing event, using CRISPR technology. Without such a more specific experiment, I think the mouse knockout does not provide strong evidence for the authors’ hypothesis.

**We agree that the ‘icing on the cake’ experiment would be to use CRISPR technology to specifically prevent the splicing event, but concluded that this experiment is outside of the timeframe of the current study, and may be unfeasible. Potential targets (tg cct cta ctt ccc cag * TTT (AGG), or in the reverse orientation: CC ATA TCC ATA GTC CCT AAA * ctg (ggg) cut next to the critical splice sequence, but unfortunately not in it., much reducing the chances of splice site removal. Arguably we could use two CRISPRs, but this is a major undertaking, as both would need to work and the deletion occur on both chromosomes. This double approach could take many months (at best), and (at worst) may not work.**

**However, as described above (point (2) we have now blocked CRB2S in dmNes+RG, and show that this prevents collapse (new Figure 7O-U), adding to the evidence that supports our model. Our model is that the interaction of CRB2S with CRB2 leads to specific changes in cell polarity that initiate cell delamination that are not triggered through genetic loss of transmembrane Crb2 in VL cells. For instance, genetic loss of transmembrane Crb2 does not lead to the characteristic loss of Par3 detected in delaminating cells.We clarify these points, and the additional points that suggest a role for CRB2S from dmNes+RG in the Discussion.**

**To strengthen evidence for the specificity of this effect (to dmNes+RG) we have also performed an additional experiment targeting the dmNes+ RG cell using an shCrb2 construct. We show that shCrb2 prevents collapse whereas a control construct does not interfere with collapse (new Supp.Fig.10). It is worth noting, however, that the combination of electroporation and long-term culture leads to rather unhealthy-looking slices – and so we present this as a supportive experiment for the conclusions drawn through the bead experiment.**

Minor points:

4. Fig 1. Diagrams at the bottom are not referred to in the legend.

**Thank you – we now refer to the diagram**

5. Fig 2. Sentence: “(EH) Immunolabelling reveals ventral and dmNes+RG that elongate as collapse proceeds …” does not make sense. Please explain what red line indicates in part I, and mention/give labels to the diagrams to make clear which data each is summarising.

**We thank the reviewer for pointing this out and now provide a better explanation in the Figure legend**

6. Fig 3. Title mentions ‘ratcheting’, but it is not clear from reading the legend what is the evidence in the data for this phenomenon. The link to ratcheting needs to be made (see also point 1 above).

**Please see response to point 1 above: Figure 3B/B’/C/C’ shows new data, where ratcheting is clear.**

7. Fig 5. Please explain in the legend what M2 means. The site of the beads in parts O,P,Q,R should be shown (e.g. by dotted lines). Similarly in Suppl Fig 8. Is the asterisk in D indicating a bead? Please explain in the legend.

**We thank the reviewer for these suggestions for clarification, all of which have been addressed and/or explained.**

8. “High power” is used throughout to refer to images. Presumably the authors mean “high magnification”?

**Thank you – we have amended this**

9. The paper title within the reference Sevc et al, 2009 is incorrect.

**Thank you – we have amended this**
