# Supplementary material for: Crumbs2 mediates ventricular layer remodelling to form the spinal cord central canal
Source: PLoS Biol. 2020 Mar 9;18(3):e3000470. doi: 10.1371/journal.pbio.3000470 (PMC7108746; doi:10.1371/journal.pbio.3000470)
Supplement: Attachment — Submitted filename: Reviewer response.docx [file pbio.3000470.s028.docx]

**Reviewer #1:**

*1a) The discussion of the mechanism of CRB2S action is rather confused. On the one hand, the authors state that  "however, dmNes+RG also secrete CRB2S, which acts non cell-autonomously and locally to compete away CRB2 in neighboring cells, leading to loss of apico-basal polarity. Together these findings add to the evidence that the fly regulatory network is conserved in vertebrates." On the other hand, they show that loss of all CRB2 isoforms in the VL cells does not lead to a loss of polarity or delamination and conclude that "Our studies suggest that the CRB2S-CRB2-mediated polarity changes exert specific downstream effects to enable delamination, which are not triggered simply through genetic loss of full-length CRB2". This seems to indicate to me that CRB2 is not required for polarity in the VL cells and that CRB2S must signal through some other mechanism.*

With respect, the reviewer has misunderstood. As we stated in the manuscript, the genetic loss-of-function experiment does not remove the secreted variant (Crb2S) We have altered the wording in places to clarify this. Additionally we have made some changes to the Discussion to clarify the different changes to polarity observed after loss of CRB2TM versus after the interaction of CRB2S-CRB2TM.

Given the importance of clearly conveying whether we are referring to Crb2TM or CRB2S we have made some additional minor amendments so that it is obvious when we are talking about *Crb2* (gene), or the two forms of the protein. These are track-changed.

*1b) They are also confused in their description of the literature of CRB2 stating that: 
disruption of CRB2-CRB2 leads to a loss of polarity and disrupted epithelial-mesenchymal transition (EMT). Ramkumar showed that Crumbs is essential in the primitive streak  for EMT (which is a loss of epithelial polarity), so this is the opposite of its disruption leading to a loss of polarity.  I suspect that that they are trying to shoehorn their results to fit the consensus model that CRB2 is required for polarity in vertebrate epithelia as it is in flies, whereas their data and Ramkunar's data suggest the opposite, i.e that it is required for a loss of polarity and EMT.*

We apologise: in trying to cut back on words, we spliced together two sentences and two references and the meaning became confused. We have clarified this section.

At the same time, the reviewer’s comments suggest that we need to be more clear about the effects of Crb2S (versus Crb2TM) on polarity, so we have made some additional minor amendments to the Discussion. In particular we have removed some of the details from the opening paragraph of the Discussion, as these were (already) expanded upon in a later part of the Discussion.

*2) I still find the MDCK data unsatisfactory. The effect on adhesion is clear, but the loss of polarity is not really obvious.*

It is not entirely clear why the reviewer finds the MDCK unsatisfactory, but we interpreted it as meaning that he/she was not satisfied with the Zo-1 data. To strengthen our evidence that exposure to Crb2S reduces polarity we have (a) chosen better representative images of Z-1 localisation; (2) performed quantitative analyses of Zo-1 distribution in MDCK cells exposed to control or CRB2S media. Additionally we now cite further previous studies, showing that MDCK cells are polarised (Shin et al., 2005).

*Reviewer #2: The authors have greatly improved the manuscript in the revised version, and are to be congratulated on providing the additional evidence for the action of CRB2S from the new blocking antibody and shCrb2 implantation studies. Additionally, Fig 3 showing the movie frames of delamination of VL cells relative to the dmNes+RG cell is now very much clearer than the corresponding figure in the original version.

I would only now request some final improvements to the documentation of the findings and experimental procedures, in the figure legends and Methods, as follows:

Fig 2. The schematics on the left and in the centre are not give labels or specifically explained in the legend. Please make this change.*

We have now labelled the schematics

*Fig 2E-H. This magnification seems too low to clearly show individual Nestin+ cells. Please indicate by arrows where the cells are on the sections, which the legend refers to.*

We have added arrows

*Fig 2J-L''. Please explain in the legend what the dotted lines and arrowheads refer to.*

We have explained this

*Fig 2N. Please explain what the insets of boxed regions show. I do not think the "dorso-ventrally oriented Sox2(+) nuclei" are clear without arrows.*

We have explained the insets and added arrows

*Fig 5H-L. The Results comments on "… Nkx6.1(+) progenitor cells appeared disorganised, the basement membrane showed breaks …". Please indicate these by arrows in the figure and include mention of these in the legend.*

We have made the necessary changes

*Fig 7. Part U is not labelled as such.*

Thank you – this is now labelled

*Methods. I think more detail is needed of the specialised in vivo procedure that has been done. The authors say they grafted dmNes+RG or VL cells, but there is very little detail on how such cells were isolated in pure form for grafting. The Methods says: "Tissue to be transplanted was punched out with a pulled glass needle (1mm x 0.78, Harvard Apparatus) and mouth pipette (Sigma), before being carefully placed into the most rostral part of the open neural tube." This leaves unclear what evidence the authors have that their punching out method was able to harvest dmNes+RG or VL cells, free from contamination with each other. Were tissue punches evaluated after isolation with markers to test this? Better description and/or more evidence of this is needed.*

Indeed, we evaluated sister-punches for marker expression and now include this data as an additional supplementary figure (new S6).
